# Some Points on the Etiology, Pathology and Treatment of Persistent Pyorrhea

**Published:** 1899-08

**Authors:** George T. Carpenter

**Affiliations:** Chicago, Ill.


### Some Points on the Etiology, Pathology and Treatment of Persistent Pyorrhea.

BY GEORGE T. CARPENTER, M.D., D.D.S., CHICAGO, ILL.

Abstract of paper read before the Section of Stomatology, Amer. Med. Ass'n Columbus, June, 1899.

It is not my intention to give a new name to the so-called pyorrhea alveolaris, but to call attention to that class of so-called incurable cases of pyorrhea which, after all efforts at treatment on the part of both practitioner and patient, pus continues to ooze from the pocket. I do not wish to be understood as including in this class the teeth that are ready for the forceps, having lost two-thirds or more of their natural support, but teeth that in the judgment of the operator should be saved but do not yield to his attempts to eradicate the disease. The one word that covers more than any other in the handling of pyorrhea is thoroughness; thoroughness in diagnosis, thoroughness in mechanical and surgical procedures, and thoroughness in all subsequent treatment. It is very generally known and accepted by most practitioners that the extraction of any tooth affected by pyorrhea will in time result in a permanent cure of the disease. It has also been repeatedly demonstrated that in the majority of cases where pyorrhetic teeth have been extracted and thoroughly cleansed of deposits and roots trimmed, removing all roughened parts, also the pulp removed and canals filled, and then replanted, such teeth grow firm and pus and pockets are not present. There must be some good reason for this changed condition, and I am convinced that this change is brought about by removing the exciting or irritating cause. I do not wish to be understood as not believing in constitutional predisposition to pyorrhea. I do not believe that cachexia, in some cases, will render pyorrhea incurable through malnutrition. But even in this class of cases we must not lose hope. Do something, and do it with thoroughness; break up the sameness of life; change the conditions. Tonic and alteratives are valuable, especially rest, sunshine and fresh air. Also, constitutional treatment for syphilitic or other taints may prove very beneficial. But the exciting cases are by far the most common and will be the principal theme of this paper.

There are three points in the irritating or exciting causes of persistent pyorrhea to which I wish to call attention, namely:

1st. The failure to reach, recognize and remove deposits.

2d. Infection indefinitely continued from septic pulp.

3d. Decalcification or molecular change in cementum and dentine.

The causes of failure in removing deposits are twofold. First, a failure to locate the deposits, and second, a failure to reach the deposit with any set of instruments now on the market. In pyorrhea cases that have received previous treatment and pus is still found present, we should make a careful differential diagnosis between deposits, pulp infection, and roughened spiculi.

To aid in this work I use 5 to 10 percent aqueous solution of cocain on cotton and pack firmly into the pocket, and allow it to remain for fifteen or twenty minutes; then protect the parts with a napkin, dry the surroundings and carefully remove the cotton, holding the mouth mirror in position so as to see all parts of the pocket the instant the cotton is removed. In cases where a better view is required, pack with antiseptic gauze and allow to remain two or three days. If deposits are seen, remove them if you can.

To insure success in removing deposits I use a pyorrhea model of thirty-two natural teeth, set in rubber tubing and arranged in upper and lower set in articulator, with a heavy rubber band in front which acts as lips, and all tooth surfaces and pockets must be reached through the rubber lips. By fastening this model to the head-rest of the operating chair an instrument can readily be fitted by using annealed stove-pipe wire from one and one-half to two inches in length in a socket handle. Flatten the point of the wire and bend so as to reach the required spot or surface. Then bend or make an instrument of the same angle, with spoon-shaped point, and with it remove the troublesome deposit.

In this way any deposit, in any location, can be reached and removed. The instruments should be kept sharp for the work, and the pull motion should be used, as there is danger with the push motion of dislodging and forcing a scale of calculi into the tissues, where it will be difficult to find it, and if not removed will again become attached and the disease will continue in a more aggravated form.

The rough deposits can be detected by the tactile sense, but the hard, smooth or glazed deposits can only be detected by actual sight. A true alveolar abscess does not discharge pus through a pyorrhea pocket. But it is not uncommon as a result of encroachment of pyorrhea at the apex of the root for inflammatory action to cause the death of the pulp, which becomes infected and will in turn reinfect the cleansed or treated pockets; and this state of affairs will continue or be repeated until the pulp is extirpated and the canals antiseptically cleansed and filled. Circumscribed abcesses may be the result of incased deposits caused by some local irritation and swelling which forms a barrier to the free escape of pus.

From experiments which I have been making on rabbits I find that infecting a fresh wound in the gum with pyorrhea or other pus, that the parts will continue inflamed from two to four days, and then rapidly heal; but by putting a rubber band around the tooth and pressing it under the gum, allowing it to remain and in this way establish a pocket, and then infect the wound with pus from pyorrhea or chronic ulcer, you will establish the disease, which will be self-sustaining. There have been experiments made in the human mouth, where the teeth had received little or no care but where there was no pyorrhea present, with similar results as in the rabbits, but there was a tendency to outgrow the disease without treatment. With treatment the cases yielded quickly and a cure resulted, but I think that in these cases the condition of the systems was such as to resist disease and re-establish health, and also that the exciting cause was not continued long enough or to the extent where we have deposits or other causes named in this paper.

From recent examinations for a specific alveolar pyorrhea bacillus from cultures infected by pus germs taken from pyorrhea pockets, a competent bacteriologist* has thus far been unable to find bacilli that are not found in pus from other infected traumatisms of the mouth. Yes, we mean traumatism, the same as in infected condition from a sliver in the flesh. And that is the condition that we have in pyorrhea alveolaris, with the same results, becoming chronic from long standing, as fistula, ulcers, etc., in other parts.

The condition of the apex of the root of some teeth will remind one of a condition known as absorption, and is generally acknowledged as such. But can a tissue be absorbed and still remain as *debris* in the pocket? Such is the condition found

* The author of this experiment will give a full report of his work on pyorrhea bacilli as soon as he completes his course of experiments.

in pyorrhea pockets, which can be easily proved by taking the contents of a pocket, dissolving it in hydrochloric acid; then add three times its bulk of water and filter, boil and when cold add a solution of ammonia, which will precipitate the phosphate of calcium.*

The same result is attained by rinsing a freshly extracted roughened pyorrhea root in cold water, then with a stiff brush and water brush the roughened parts and put the resulting product into a test tube; add hydrochloric acid and water, if necessary, and filter and boil; to this add a solution of ammonia and the lime salts are precipitated. This decalcified cementum and dentine, through their roughened surfaces, spiculi and *debris*, act as irritants to the already inflamed tissues which are in the depth of the pocket, and as a result pus will continue to flow.

Many teeth affected with pyorrhea may have a pocket only on one side of their root, leaving the three remaining sides healthy. Other teeth may have only one root affected, and the other root or roots, as the case may be, are in good condition. To illustrate I will recite some persistent cases from practice.

Case 1.—Mr. G., about thirty years of age; employment indoors and very confining. The color of the skin and mucous membrane would suggest anemia. Lacerations or injuries to soft parts are slow to heal. He had very serious trouble after the removal of an impacted third molar. In 1898 I made an examination of his mouth and found the right upper central and left lower central affected, having deep anterior pyorrhea pockets with a profuse flow of pus. I gave both cases thorough surgical treatment, which consisted in the removal of all deposits and the cureting of pockets and margins of the process. I then filled the pockets with iatrol which had been moistened with equal parts of oil of cassia and carbonate of creosote, and painted the gums with tincture of iodin, repeating the iodin treatment about twice a week.† He derived little, if any, benefit from this treatment. I then put the patient on tonic and alterative treatment, and in

* Atfield's General Medical and Pharmaceutical Chemistry, Calcium Reaction.

† Dr. Talbot, Pyorrhea Alveolaris, *International Dental Jorunal*, April, 1896.

about three months the pus in the pocket of the upper central disappeared and the pocket closed.

About this time I drilled into the lingual surface of the lower central and found absence of the pulp.

I used thorough antiseptic treatment of canal and filled the same with chloral-percha and gutta percha points. Treatment was continued from once to twice a week for two months longer, but pus still persisted. In June I examined apex of root and found it denuded and roughened. I amputated the lower fourth of the root and rounded the stump; the soft parts healed kindly under antiseptic treatment, and no pus has been present at any time since the operation. The gums are not yet entirely restored, but the tooth is doing good service.

Case 2.—Mr. H., about fifty years of age. Has given his teeth considerable care, and I think caused pyorrhea by injudicious use of the wooden toothpick. Has had several teeth affected by and treated for pyorrhea during the last ten years, and in all but two cases a cure has been effected. In June, 1898, he was referred to me by a brother practitioner, and on examination I found the upper right second bicuspid and first and second upper right molars, also the interproximal space between right lower second bicuspid and first lower molar, also lower central on same side, diseased.

He had an old chronic pocket on anterior root of left lower first molar. This tooth and the first upper molar on the opposite side had been treated by a good dentist and given up as hopeless about eight years previous, and the patient was instructed to use a syringe with an antiseptic to keep the pockets clean, as the best that could be done for them. I gave the usual surgical treatment, as in the former case, for the four other teeth, which yielded readily and a cure was effected.

At my solicitation the gentleman allowed me to experiment with the two chronic cases. On examination I found the pulps dead in both teeth, which were removed and the canals filled. I then gave them both a most thorough surgical treatment, which resulted in the almost entire cessation of pus for a short time, but the old condition was resumed. I re-examined the pockets for cause and continued antiseptic and stimulating treatment until September, when I considered the case hopeless and decided to amputate the roots.

In the upper molar I removed the anterior buccal root. The soft parts closed up and yielded readily to treatment, and we have not had the appearance of pus up to the present time. After becoming satisfied with the result in the upper molar, I amputated the anterior root of the lower molar with similar results. The teeth are now doing good work with an absence of pus. The roots were found roughened and slight nodules of cementum around the apices showing nature's attempt to recalcify and thus produce a cure. Many other cases could be reported to show the success following the thorough removal of the cause for persistence of pus in pyorrhea, which to my mind proves the local character of the disease and that malnutrition plays but a small part when the actual irritation is removed.

